# Association of Mean Platelet Volume With Vascular Complications in the Patients With Type 2 Diabetes Mellitus

**DOI:** 10.7759/cureus.29316

**Published:** 2022-09-19

**Authors:** Krutik J Brahmbhatt, Bharat Chaudhary, Darshankumar M Raval, Shashwat Mallik, Shahin Khan, Mayur Patel, Nirav Patel

**Affiliations:** 1 Internal Medicine, Medical College Baroda, Vadodara, IND; 2 Medicine, Medical College Baroda, Vadodara, IND

**Keywords:** mean platelet volume (mpv), macrovascular and microvascular complications, glycated hemoglobin (hba1c), glycemic status, diabetes mellitus type 2

## Abstract

Background

Diabetes mellitus (DM) is a global health concern that is predicted to involve over 10% of the adult population by the next decade. Vascular complications are the major source of mortality and morbidity in diabetics. Mean platelet volume (MPV) which indicates platelet activity may play a crucial role in the vascular effects of DM and, hence, can be used as a prognostic marker. We have attempted to study the association of MPV with the glycemic status, duration of diabetes, and presence of vascular complications in diabetics.

Methods

A cross-sectional study of 300 patients with type 2 DM aged ≥18 years admitted to the inpatient department of medicine was carried out in a tertiary care hospital. After subgrouping patients according to their glycemic status and MPV, the association between microvascular and macrovascular complications was studied.

Results

The majority of patients were >60 years of age and an increasing prevalence of vascular complications was noted with increasing age. Forty-six percent and 45% of the patients had microvascular and macrovascular complications, respectively. Ischemic heart disease (IHD) and retinopathy were the most common macrovascular and microvascular complications, respectively. Vascular complications in DM showed a significant association with MPV, fasting blood sugar (FBS), post-prandial blood sugar (PP2BS), glycated hemoglobin (HbA1c), and the duration of diabetes.

Conclusion

A high MPV was linked to poor glycemic control, a longer duration of diabetes, and an increased prevalence of vascular complications. Hence, MPV could be used as a cost-effective marker to predict vascular complications in patients with type 2 DM.

## Introduction

Diabetes mellitus (DM) is a rapidly growing global health issue, which is a result of a problem with insulin secretion or its effect on the body. It is estimated to involve over 10% of the adult population by the end of the next decade, out of which a large chunk remains unaware of their diagnosis. Initially believed to be a lifestyle disease in the developed world, DM has now established itself in developing nations as well with three out of four adults with diabetes living in low to middle-income countries [1,2]. DM is characterized by chronic hyperglycemia due to the lack of insulin or its function, which causes endothelial dysfunction and negatively impacts vascular structures over the long term [3]. The vascular complications of type 2 DM are divided into microvascular and macrovascular complications, and both have shown an association with the glycemic control of patients. While atherosclerosis is one of the main causes of early mortality in DM, microvascular complications in DM like nephropathy and retinopathy are major contributors to chronic kidney disease and blindness worldwide [4]. These complications place a huge burden on the healthcare systems of countries, especially in developing nations like India.

Platelets are blood cells that are primarily involved in hemostasis by the formation of the primary platelet plug, which further leads to thrombus formation. Larger platelets are more active and contain more dense granules which result in a stronger procoagulant effect and more thrombosis. Hence, the mean platelet volume (MPV) may have a causal association with the vascular complications seen in type 2 DM. But on the other hand, MPV may also be raised as a consequence of thrombotic events due to the rapid consumption of small platelets and rushed production of reticulated platelets by the bone marrow [5]. In either case, MPV could be used as a biomarker for vascular complications in type 2 DM. This could also be helpful as a cost-effective prognostic marker for type 2 DM in resource-limited settings. Apart from DM, a raised MPV is also seen in several cardiovascular and cerebrovascular events and intestinal diseases like Crohn's disease. Similarly, a low MPV has also been described as a marker in diseases such as tuberculosis, ulcerative colitis, and several carcinomas [6].

In physiological conditions, a lower MPV corresponds to a higher platelet count and vice versa. But in pathologies with inflammation, there is a marked increase in thrombopoietin generation and activation of megakaryocytes by cytokines such as IL-6 which leads to a high platelet count along with large platelets. Hence, MPV has been suggested as a prognostic marker in various cardiovascular and cerebrovascular diseases as increased platelet activity leads to thrombotic events in susceptible individuals [6].

This study attempts to observe the correlation of MPV with vascular complications in diabetic patients. We have tried to determine whether platelets are activated in diabetes and its associated vascular complications by comparing the MPV between patients with vascular complications and those without them. The correlation of MPV with the glycemic status and duration of diabetes has also been studied. Additionally, we have attempted to observe the most common microvascular and macrovascular complications among diabetic patients.

## Materials and methods

A cross-sectional study was conducted in the department of medicine of a tertiary care hospital after taking due approval from the Institutional Ethics Committee of Biomedical and Health Research (#IECBHR/19-2021). After obtaining informed consent, the first 300 patients with type 2 DM (according to American Diabetes Association criteria) aged ≥18 years in the inpatient department of medicine were enrolled in the study [7]. Only patients who had been diagnosed with type 2 DM before the onset of signs and symptoms of vascular complications were included. Pediatric patients (<18 years), those with an abnormal platelet count (<100,000 or >400,000 platelets per microliter), those using drugs affecting platelet function, those with severe anemia (hemoglobin <8.0 g/dL) or hemoglobinopathies, pregnant females, or patients with acute or chronic infections, and chronic diseases or malignancies were excluded [8]. A detailed history was taken for each patient and a clinical examination was done for vascular complications.

The laboratory investigations done for each patient included a complete hemogram with MPV, blood urea, serum creatinine, urine routine microscopy, microalbuminuria test, coagulation profile, random blood sugar (RBS), fasting blood sugar (FBS), post-prandial blood sugar (PP2BS), glycated hemoglobin (HbA1c), echocardiography, lipid profile, and fundoscopy, while some other investigations like two-dimensional echocardiography, ultrasonography of kidney-ureter-bladder (USG-KUB), neuroimaging, and nerve conduction velocity study were done only if required. We measured the MPV and platelet counts with a complete blood count using an automated blood analyzer. The estimation of plasma glucose levels (FBS, PP2BS) was carried out by the glucose-oxidase method and that of HbA1c by the high-performance liquid chromatography method. After baseline evaluation, patients were sub-grouped according to their glycemic status and MPV, and their association with microvascular and macrovascular complications was studied.

Since the data was largely qualitative, the chi-square test was used. The analysis of data was done using MedCalc software (Ostend, Belgium: MedCalc Software Ltd.) and a p-value of <0.05 was considered statistically significant. receiver operating characteristic (ROC) curve analysis for age, FBS, HbA1c, and MPV for the diagnosis of vascular complications has been done using Microsoft Excel.

## Results

Out of 300 patients with type 2 DM, there were 153 males and 147 females with a mean age of 53.96±12.17 years. The majority of the patients (35%) were >60 years of age while only 16% were aged <40 years. Majority of the patients had a FBS <200 mg/dL, PP2BS >350 mg/dL, HbA1c more than eight and MPV ≤12 fL (Table 1).

**Table 1:** Distribution of patients according to fasting blood sugar, post-prandial blood sugar, glycated hemoglobin, mean platelet volume, and duration of diabetes. FBS: fasting blood sugar; PP2BS: post-prandial blood sugar; HbA1c: glycated hemoglobin; MPV: mean platelet volume

Variable	Number of patients
FBS (mg/dL)	
<200	123
200-250	114
251-300	42
>300	21
PP2BS (mg/dL)	
<250	48
251-300	72
301-350	60
>350	120
HbA1c	
<8	39
8.1-10	144
10.1-12	66
>12	51
MPV (fL)	
8-10	123
10-12	126
>12	51
Duration of diabetes	
<5 years	114
5-10 years	75
>10 years	111

Microvascular complications were present in 138 patients while macrovascular complications were recorded in 135 patients. Among microvascular complications, retinopathy was the most common (43%), followed by neuropathy (37%) and nephropathy (20%). Among macrovascular complications, ischemic heart disease (IHD) was the most common (41%), followed by cerebrovascular accidents (CVA) (36%) and peripheral vascular disease (PVD) (23%).

While gender did not have any association with the presence of vascular complications, a significant rise in both microvascular and macrovascular complications was noted with increasing age (p <0.001) (Table 2).

**Table 2:** Association of microvascular and macrovascular complications with sociodemography.

Variables	Microvascular complication		p-Value
	Yes	No	
Age-group			
<40 years	3	45	<0.001
40-50 years	9	54	
51-60 years	39	36	
>60 years	87	27	
Gender			
Male	72	81	0.7073
Female	66	81	
Variables	Macrovascular complication		p-Value
	Yes	No	
Age-group			
<40 years	0	51	<0.001
40-50 years	3	63	
51-60 years	42	36	
>60 years	90	15	
Gender			
Male	66	87	0.5082
Female	69	78	

Significant associations were found between microvascular complications and increasing FBS, PP2BS, HbA1c, MPV, and duration of diabetes (Table 3). Macrovascular complications were also significantly correlated with these parameters (Table 4). Out of the 117 patients having a high HbA1c (>10), 51 had an MPV >12 fL while 66 had an MPV ≤12 fL. In the subgroup of MPV >12 fL (HbA1c >10), 45 out of 51 patients had microvascular complications and 39 out of 51 patients had macrovascular complications (p >0.05). In the subgroup of MPV ≤12 fL (HbA1c >10), 39 out of 66 patients had microvascular complications, and 42 out of 66 patients had macrovascular complications (p >0.05).

**Table 3:** Association of microvascular complications with fasting blood sugar, post-prandial blood sugar, glycated hemoglobin, mean platelet volume, and duration of diabetes. FBS: fasting blood sugar; PP2BS: post-prandial blood sugar; HbA1c: glycated hemoglobin; MPV: mean platelet volume

Variables	Microvascular complication		p-Value
	Yes	No	
FBS (mg/dL)			
<200	30	93	<0.001
200-250	60	54	
251-300	33	9	
>300	15	6	
PP2BS (mg/dL)			
<250	9	39	<0.001
251-300	21	51	
301-350	24	36	
>350	84	36	
HbA1c			
<8	3	36	<0.0001
8-10	51	93	
10.1-12	39	27	
>12	45	6	
MPV (fL)			
8-10	21	102	<0.0001
10-12	72	54	
>12	45	6	
Duration of diabetes			
<5 years	6	108	<0.0001
5-10 years	39	36	
>10 years	93	18	

**Table 4:** Association of macrovascular complications with fasting blood sugar, post-prandial blood sugar, glycated hemoglobin, mean platelet volume, and duration of diabetes. FBS: fasting blood sugar; PP2BS: post-prandial blood sugar; HbA1c: glycated hemoglobin; MPV: mean platelet volume

Variables	Macrovascular complication		p-Value
	Yes	No	
FBS (mg/dL)			
<200	24	99	<0.0001
200-250	66	48	
251-300	27	15	
>300	18	3	
PP2BS (mg/dL)			
<250	6	42	<0.0001
251-300	21	51	
301-350	21	39	
>350	87	33	
HbA1c			
<8	9	30	<0.0001
8-10	45	99	
10.1-12	36	30	
>12	45	6	
MPV (fL)			
8-10	27	96	<0.0001
10-12	69	57	
>12	39	12	
Duration of diabetes			
<5 years	3	111	<0.0001
5-10 years	30	45	
>10 years	102	9	

**Figure 1:**
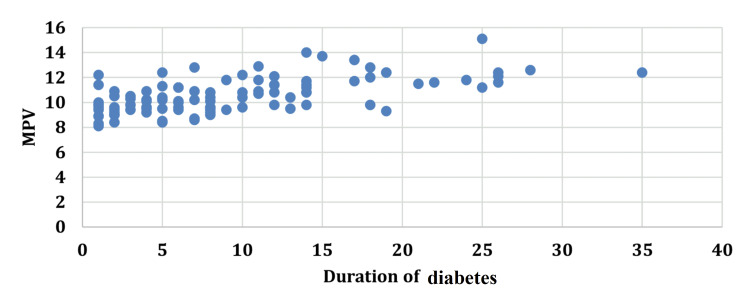
Correlation between duration of diabetes and mean platelet volume. MPV: mean platelet volume

**Figure 2:**
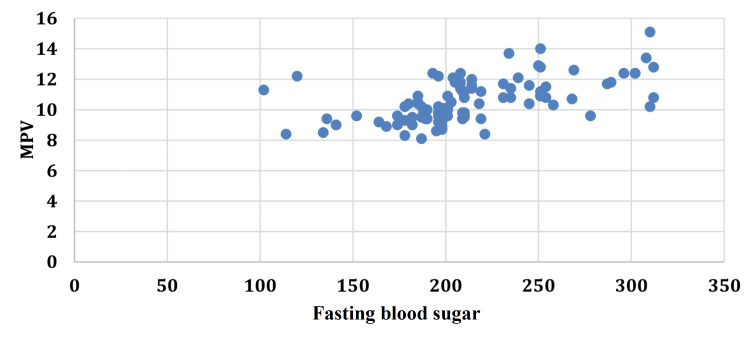
Correlation between fasting blood sugar and mean platelet volume. FBS: fasting blood sugar; MPV: mean platelet volume

**Figure 3:**
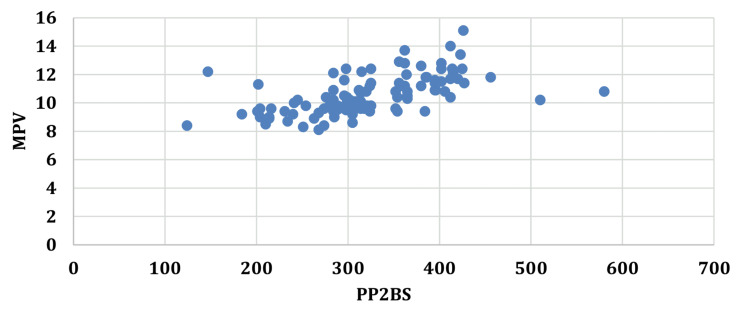
Correlation between post-prandial blood sugar and mean platelet volume. PP2BS: post-prandial blood sugar; MPV: mean platelet volume

**Figure 4:**
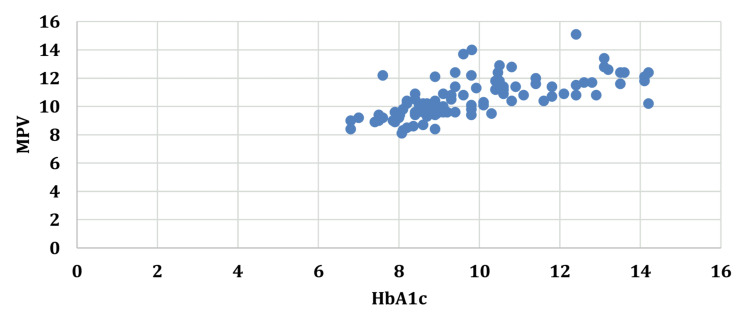
Correlation between glycated hemoglobin and mean platelet volume. HbA1c: glycated hemoglobin; MPV: mean platelet volume

An increase in the duration of diabetes was accompanied by an increase in the MPV with a correlation coefficient (r) of 0.6521. A similar correlation of MPV was seen with FBS (r=0.5649), PP2BS (r=0.5712), and HbA1c (r=0.6627) (Figures 1-4).

ROC analysis of age, FBS, HbA1c, and MPV for the diagnosis of both microvascular and macrovascular complications was done (Table 5, Figures 5-12).

**Table 5:** Receiver operating characteristic analysis with area under the curve. AUC: area under the curve; FBS: fasting blood sugar; HbA1c: glycated hemoglobin; MPV: mean platelet volume

Microvascular complications					Cut-off	Macrovascular complications				
Total AUC	AUC	Youden index	Specificity	Sensitivity		Sensitivity	Specificity	Youden index	AUC	Total AUC
Age (years)										
0.7712	0.2320	0.0432	0.0432	1	>30	1	0.0303	0.0303	0.2787	0.8660
	0.3152	0.2560	0.2777	0.9782	>40	1	0.3090	0.3090	0.3775	
	0.1714	0.5241	0.6111	0.9130	>50	0.9777	0.6909	0.6686	0.1793	
	0.0523	0.4637	0.8333	0.6304	>60	0.666	0.9090	0.5757	0.0303	
FBS (mg/dL)										
0.6927	0.4896	0.0246	0.0246	1	>90	1	0.0606	0.0606	0.4914	0.6819
	0.1884	0.3566	0.5740	0.7826	>200	0.8222	0.6000	0.4222	0.1680	
	0.0126	0.2552	0.9074	0.3478	>250	0.3333	0.8909	0.2242	0.0212	
	0.0020	0.0716	0.9629	0.1086	>300	0.1333	0.9828	0.1151	0.0012	
HbA1c										
0.7470	0.2075	0.0123	0.0123	1	>6.4	1	0.0121	0.0121	0.1640	0.7149
	0.4555	0.2004	0.2004	0.9782	>8	0.9333	0.1818	0.1151	0.4600	
	0.0778	0.4049	0.4049	0.6086	>10	0.6000	0.7818	0.3818	0.0848	
	0.0060	0.2890	0.2890	0.3260	>12	0.3333	0.9636	0.2969	0.0060	
MPV (fL)										
0.7834	0.5817	0	0	1	>8	1	0	0	0.5236	0.7222
	0.1956	0.4774	0.6296	0.8478	>10	0.8000	0.5818	0.3818	0.1880	
	0.0060	0.2890	0.9629	0.3260	>12	0.2888	0.9272	0.2161	0.0105	

**Figure 5:**
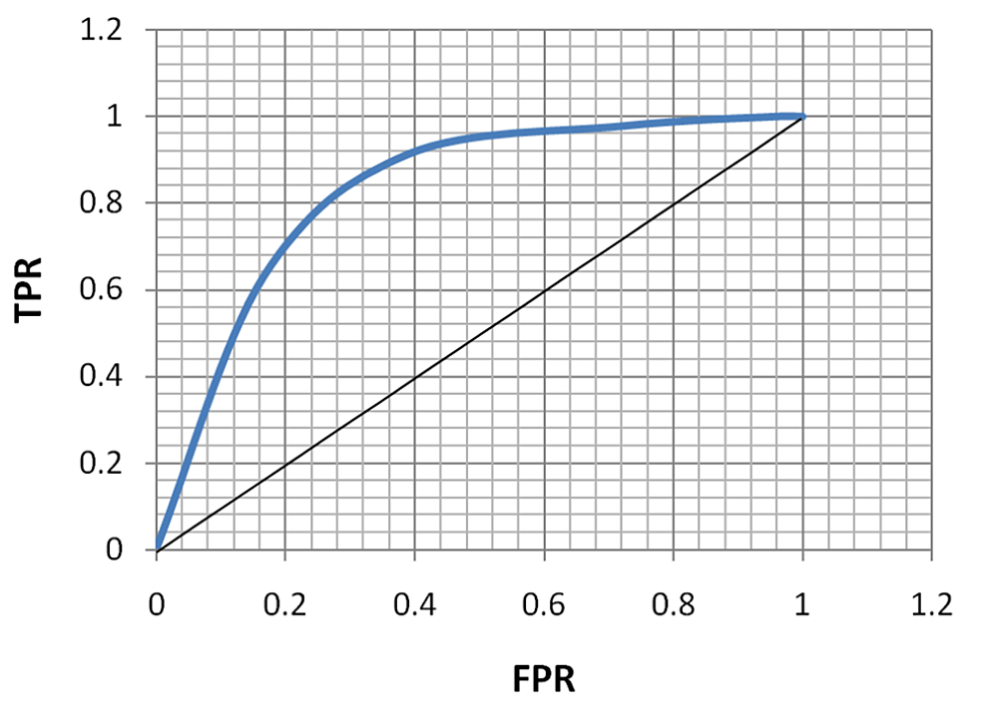
Receiver operating characteristic curve for age in diagnosing microvascular complications. TPR: true positivity rate; FPR: false positivity rate

**Figure 6:**
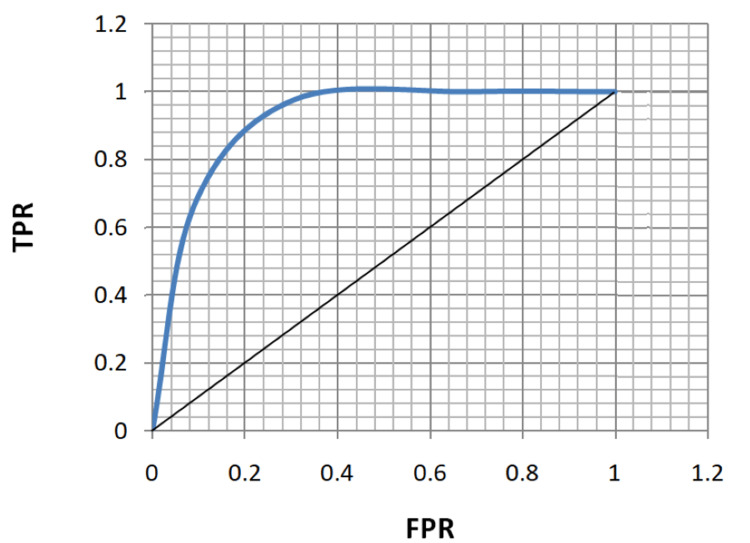
Receiver operating characteristic curve for age in diagnosing macrovascular complications. TPR: true positivity rate; FPR: false positivity rate

**Figure 7:**
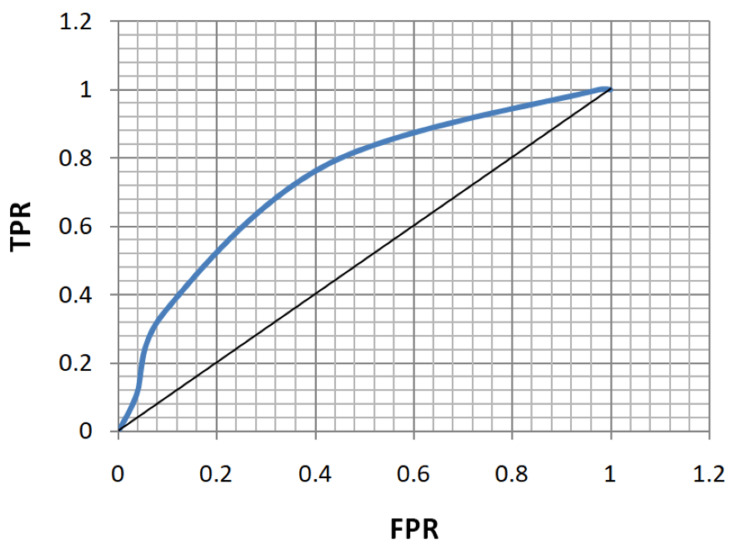
Receiver operating characteristic curve for fasting blood sugar in diagnosing microvascular complications. TPR: true positivity rate; FPR: false positivity rate

**Figure 8:**
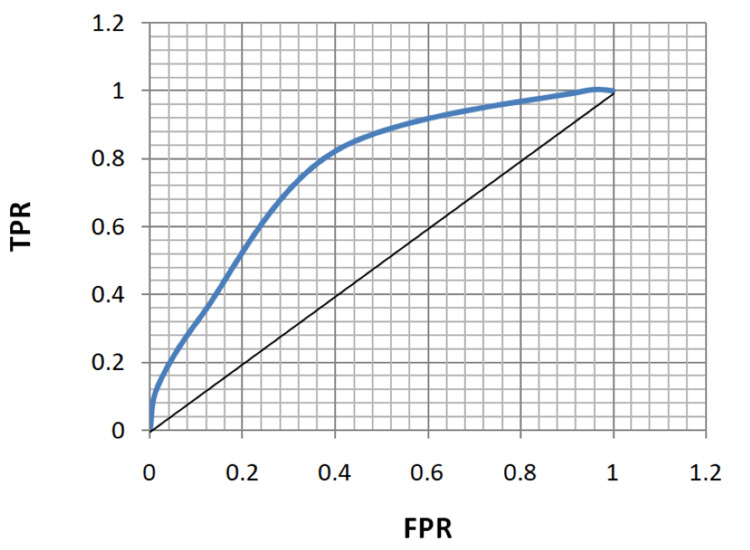
Receiver operating characteristic curve for fasting blood sugar in diagnosing macrovascular complications. TPR: true positivity rate; FPR: false positivity rate

**Figure 9:**
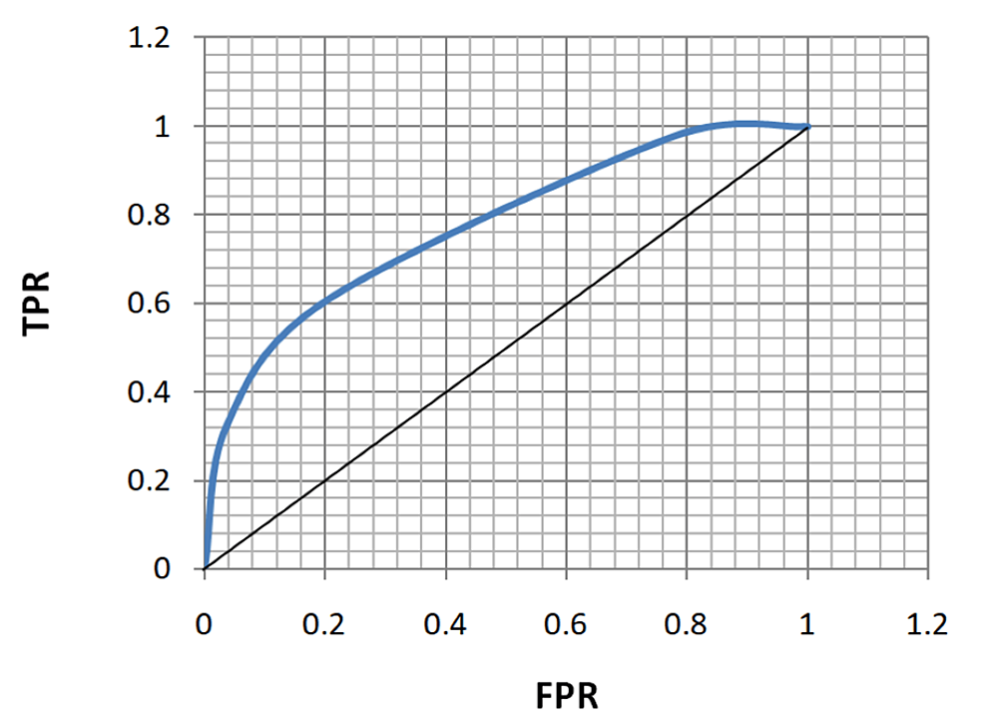
Receiver operating characteristic curve for glycated hemoglobin in diagnosing microvascular complications. TPR: true positivity rate; FPR: false positivity rate

**Figure 10:**
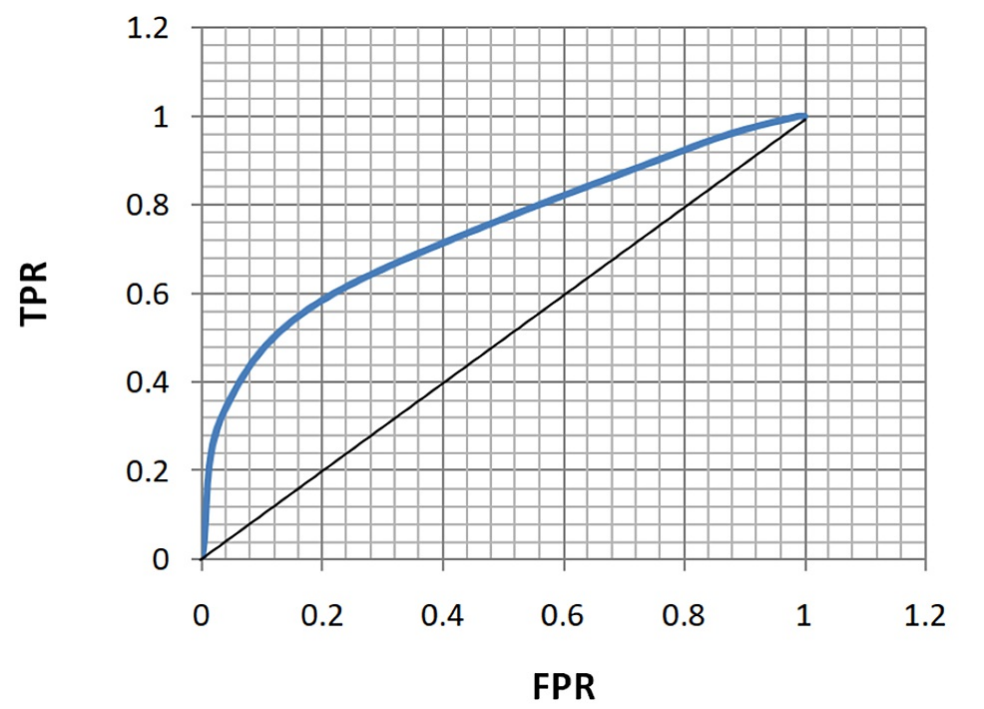
Receiver operating characteristic curve for glycated hemoglobin in diagnosing macrovascular complications. TPR: true positivity rate; FPR: false positivity rate

**Figure 11:**
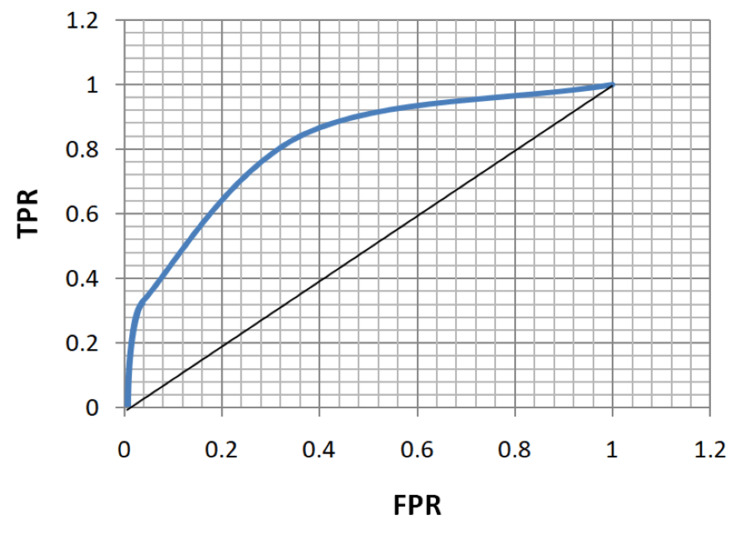
Receiver operating characteristic curve for mean platelet volume in diagnosing microvascular complications. TPR: true positivity rate; FPR: false positivity rate

**Figure 12:**
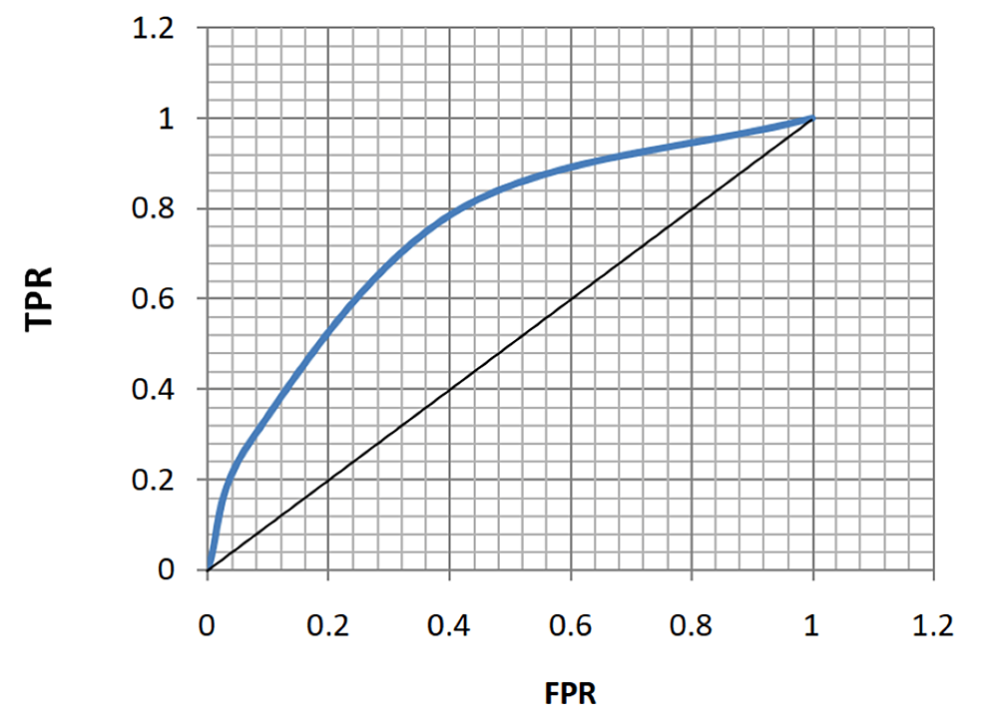
Receiver operating characteristic curve for mean platelet volume in diagnosing macrovascular complications. TPR: true positivity rate; FPR: false positivity rate

## Discussion

The most morbid complications of type 2 DM are vascular, which severely affect the life span as well as the quality of life in patients, along with increasing the health expenditure for complications that can be easily prevented by intensive glycemic control [1,4]. The economic burden of the disease is detrimental to middle-income countries like India, which is expected to remain a major contributor to the disease in the future, and, hence, early detection and prognosis of these vascular events with accessible and affordable biomarkers is the need for the hour [9]. This study attempts to evaluate the glycemic status and MPV as potential biomarkers for vascular complications in type 2 DM.

A study from Saudi Arabia found that microvascular complications like retinopathy, nephropathy, and neuropathy were present in 15%, 14.8%, and 5.6% of diabetic patients, respectively, while macrovascular complications like IHD and CVA were present in 8% and 6% patients, respectively [10]. A study from South India reported the prevalence of microvascular and macrovascular complications to be 52.1% and 29.7%, respectively. Neuropathy was the most common microvascular complication and coronary artery disease and PVD were the most common macrovascular complications [11]. Another study from Sudan reported nephropathy to be the most common microvascular complication among type 2 diabetics [12]. In contrast, our study found that microvascular and macrovascular complications were present in 46% and 45% of diabetic patients, respectively. Retinopathy and IHD were the most common microvascular and macrovascular complications in this study. Similar to our study, advancing age is associated with more vascular complications [13-16]. Some studies have shown that elderly males with type 2 diabetes have a higher rate of IHD than similarly aged females, and female diabetics are more prone to diabetic retinopathy than their male counterparts [14,16]. But no such association of vascular complications with gender was found in our study. A higher risk of vascular complications has been attributed to poor glycemic control and a longer duration of diabetes, which is in concordance with our results [13,17,18]. A study intended to identify the association between diabetic vascular complications with the duration of DM supported our findings (hazard ratio=1.3, confidence interval=1.08 to 1.17) [17]. A study used FBS >152 mg/dL as the criterion for poor glycemic control, which had a positive association with the prevalence of diabetic complications [18].

Increased platelet activity has been hypothesized to be a crucial factor in the development of vascular complications in type 2 DM. A marker of platelet function, the MPV has been widely used to study platelet activation in the disease. Younger and more active platelets which can easily aggregate have a higher MPV, while lesser active platelets have lower MPV [5,19,20]. Several studies have shown a significant association of MPV with FBS, PP2BS, and HbA1c which is in concordance with our results [21-23]. A study aimed at seeing the correlation between MPV and blood glucose level after glucose loading in normoglycemic and prediabetic patients suggested that there was a positive correlation between MPV and the post-challenge glucose level after two hours [24]. A meta-analysis of MPV levels in diabetic retinopathy revealed that MPV levels were significantly higher in patients with diabetic retinopathy as compared to those without diabetic retinopathy [25]. Our study found that higher values of MPV were associated with a higher prevalence of microvascular and macrovascular complications, which is similar to other studies [26,27]. But, on stratification of MPV with high HbA1c (>10), the significant association with vascular complications was lost. Additionally, a high MPV is not only associated with vascular complications in type 2 DM but also in non-diabetics. The increased thrombotic potential due to a raised MPV has resulted in worse outcomes in acute ischemic stroke patients. It has also emerged as an independent risk factor for myocardial infarction. In coronary artery disease patients with a high MPV, the baseline thrombogenicity is increased and in case the atherosclerotic plaque ruptures, the large platelets rapidly form a thrombus leading to occlusion of the artery [28,29]. Thus, MPV may be crucial in predicting vascular complications even in diabetics with satisfactory glycemic control.

Since the sample population was largely from Western and Central India, a multicentric prospective study should be conducted for the generalizability of these results which could also include other platelet indices like platelet distribution width and platelet large cell ratio. All patients in this study were indoor cases and, hence, most had poor glycemic control and diabetic complications. Consequently, the number of patients with good glycemic control was very less and the prevalence of vascular complications may be falsely inflated. Though hyperglycemia may play a role in the prothrombotic state, the independent association of MPV with vascular complications should not be ruled out as this association has also been seen in those without type 2 DM. While the association of MPV with vascular complications in type 2 DM should be unquestionable, the cross-sectional nature of this study further prevents determining whether raised MPV is a causative factor for vascular complications including atherosclerosis, or is a byproduct of them.

## Conclusions

Poor glycemic control indicated by a high FBS, PP2BS, or HbA1c along with a longer duration of diabetes were all linked to a higher risk of vascular complications in patients of type 2 DM. A high MPV was also found to be significantly associated with microvascular and macrovascular complications and also has a strong correlation with FBS, PP2BS, HbA1c, and duration of diabetes. Hence, it could be used as a cost-effective marker to monitor for vascular complications in type 2 diabetic patients, especially in limited-resource settings. Even though MPV has a significant association with vascular complications, stratification of MPV with high HbA1c (>10) was not found to have a significant association with vascular complications.
